# Apelin Levels in HFrEF and Association with Sustained VT Detected by ICD Interrogation: A Retrospective Pilot Study

**DOI:** 10.3390/jcdd13020079

**Published:** 2026-02-04

**Authors:** Abdullah Eren Cetin, Mustafa Lutfullah Ardic, Fadime Koca, Hilmi Erdem Sumbul, Mevlut Koc

**Affiliations:** 1 Department of Cardiology, 25 Aralık State Hospital, Gaziantep 27060, Turkey; md.a.erencetin@gmail.com; 2 Department of Cardiology, University of Health Sciences, Adana Health Practice and Research Center, Adana 01230, Turkey; drmustafaardic@gmail.com; 3 Department of Cardiology, Cukurova State Hospital, Adana 01170, Turkey; drfadimekoca@gmail.com; 4 Department of Internal Medicine, University of Health Sciences, Adana Health Practice and Research Center, Adana 01230, Turkey; erdemsumbul@gmail.com

**Keywords:** biomarker, ICD interrogation, risk stratification

## Abstract

Introduction: The serum apelin level in patients with heart failure with reduced ejection fraction (HFrEF) and its relationship with ventricular tachycardia (VT) are not clearly known. This study aimed to investigate changes in serum apelin levels in patients with HFrEF and their relationship with VT. Method: This retrospective pilot study included 90 patients with 30 patients in each group: Group I: HFrEF with documented VT; Group II: HFrEF without VT; Group III: control group without HFrEF. In addition to routine parameters, apelin levels were measured. All parameters were compared between Group I–II–III. Parameters associated with VT were identified. Result: Apelin levels were found to be significantly lower in Group I–II than in Group III. Serum glucose, creatinine, and left atrial diameter were shown to be significantly higher in Group I–II than in Group III. HDL cholesterol and left ventricular ejection fraction (LVEF) levels were significantly lower in Group I–II compared with Group III. A positive and negative correlation was found between plasma apelin levels and LVEF and age, respectively. In logistic regression analysis, apelin levels and LVEF were found to independently determine VT (OR = 0.313, 95%CI: 0.124–0.788, *p* = 0.014 and OR = 0.912, 95%CI: 0.877–0.968, *p* < 0.001). In the ROC analysis, the cut-off value for apelin was determined to be 0.80 ng/mL, and it distinguished VT status in this sample with acceptable sensitivity and specificity. Discussion: According to the results of our study, apelin levels are significantly reduced in patients with HFrEF, and reduced apelin levels are associated with the presence of VT in these patients.

## 1. Introduction

Apelin is secreted as a 77-amino acid pre-protein, which is subsequently cleaved enzymatically into various fragments differing in size and localization, including apelin-13, -16, -17, -19, and -36 [1]. The apelin receptor, known as APJ, plays an active role in the cardiac and cardiovascular (CV) system, particularly in neurohormonal regulation [2].

In several studies, patients with heart failure with reduced ejection fraction (HFrEF), plasma apelin levels have been measured, but the results have been inconsistent and sometimes contradictory. While numerous studies have reported decreased apelin levels in HFrEF patients [3,4,5,6,7,8,9], others have found increased, unchanged, or stage-dependent apelin levels [10,11,12,13,14,15]. Some studies suggest that apelin may increase during the early stages of heart failure but decrease as the disease progresses [6,10,12].

Apelin exhibits several favorable cardiovascular properties, including vasodilatory, positive inotropic, and diuretic effects [15,16,17,18]. Due to these beneficial actions, reduced serum apelin levels in HFrEF patients may contribute to adverse cardiovascular outcomes. Nevertheless, the relationship between serum apelin levels and disease prognosis in HFrEF remains unclear. While some studies have reported a significant association between plasma apelin levels and heart failure prognosis [3,19], others have found no such prognostic value [4,20]. Patients with HFrEF who have an implantable cardioverter–defibrillator (ICD) implanted for primary or secondary prevention generally have more advanced heart failure, more severe myocardial disease, and receive more extensive medical therapy. Therefore, we hypothesized that serum apelin levels might be even more markedly reduced in this group of patients.

A major cause of morbidity and mortality in patients with HFrEF is the development of ventricular tachycardia (VT) and ventricular fibrillation (VF) [21]. To the best of our knowledge, no prior study has examined the relationship between serum apelin levels and VT occurrence in HFrEF patients.

Therefore, the aim of our pilot and hypothesis-generating study was to investigate the changes in serum apelin levels in HFrEF patients and their potential association with VT.

## 2. Materials and Methods

### 2.1. Study Population

In our retrospective pilot study, patients presented to the Arrhythmia Clinic of the University of Health Sciences Adana City Training and Research Hospital between 1 May 2020 and 1 May 2024 and met the following criteria were screened: patients with heart failure with reduced ejection fraction (HFrEF; EF ≤ 40%), with an ICD implanted for primary or secondary prevention, and under appropriate medical therapy according to their NYHA functional class (I–IV) as well as age- and sex-matched control subjects without. Serum samples were obtained from all patients prior to ICD implantation and stored at −80 °C.

**Figure 1:**
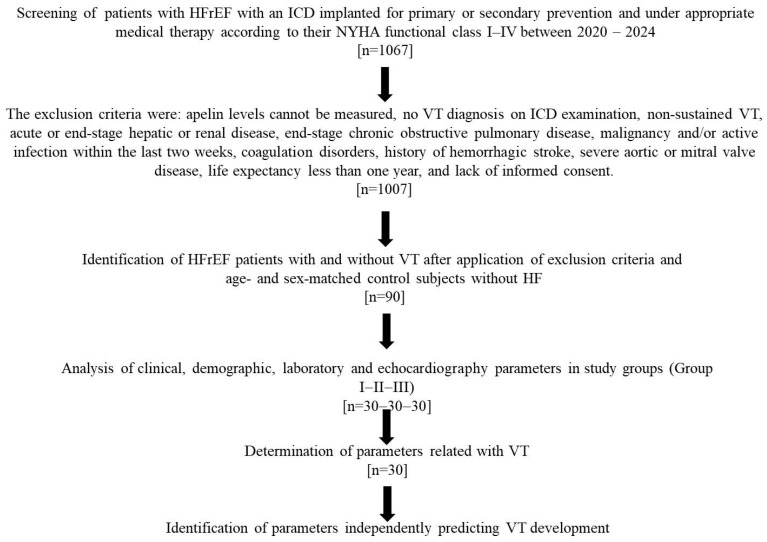
Flowchart of the study.

Prior to inclusion, each patient was independently evaluated by two cardiologists for the presence of ventricular tachycardia (VT). The presence of sustained VT (>30 s in duration or requiring shock therapy) detected by ICD interrogation after patient discharge and NYHA classification was documented. The diagnosis of VT based on ICD record interrogation was independently performed by two cardiology specialists (FK and MLA), who were blinded to each other’s assessments. In addition, although ICD programming detection zones may vary according to individual patient characteristics (such as resting heart rate, presence of atrial fibrillation, and prior VT rate), VT was defined as tachycardia episodes requiring either an initial detection of >32 beats with a tachycardia cycle length of >450 ms (133 bpm) or an initial detection of 16 beats with a redetection of 12 beats and a cycle length of <360 ms (167 bpm). Following initial screening of 1067 HFrEF patients with ICDs, and after applying exclusion criteria, 90 patients were enrolled, with 30 patients in each group: Group I: HFrEF with documented VT, Group II: HFrEF without VT, and Group III: control group without HFrEF. The study population included 62 males and 28 females, with a mean age of 61.2 ± 10.9 years. The diagnosis and management of HFrEF were based on the most recent ESC heart failure guidelines [21]. The exclusion criteria were: apelin levels cannot be measured, no VT diagnosis on ICD examination, non-sustained VT, acute or end-stage hepatic or renal disease, end-stage chronic obstructive pulmonary disease, malignancy and/or active infection within the last two weeks (diagnosis made through clinical or laboratory examinations), coagulation disorders, history of hemorrhagic stroke, severe aortic or mitral valve disease, life expectancy less than one year, and lack of informed consent. All patients were fully informed about the study and provided written informed consent. The study protocol was approved by the local institutional ethics committee. The study algorithm is shown in Figure 1.

In our study, only apelin levels were measured at the time of study inclusion. All other variables were obtained retrospectively from electronic medical records. All patients underwent detailed medical history evaluation and comprehensive physical examination. Baseline resting heart rate in sinus rhythm, systolic and diastolic blood pressure, and NYHA classification were recorded. Laboratory assessments included blood biochemistry and a complete blood count. Biochemical tests comprised glucose, urea, creatinine, total cholesterol, triglycerides, low-density lipoprotein (LDL) cholesterol, high-density lipoprotein (HDL) cholesterol, and high-sensitivity C-reactive protein (hs-CRP). White blood cell counts were also obtained.

All patients underwent M-mode and two-dimensional transthoracic echocardiography in the Echocardiography Laboratory using the EPIC 7C system (Philips Healthcare, Andover, MA, USA). Echocardiographic assessments were independently performed by two cardiology specialists who were blinded to each other’s evaluations. Left ventricular (LV) diameters were measured in the parasternal long-axis view by positioning the M-mode cursor just beyond the tips of the mitral valve leaflets and perpendicular to the LV long axis. Left ventricular ejection fraction (LVEF) and volumes were calculated using Simpson’s method from apical two-chamber (A2C) and apical four-chamber (A4C) views. Left atrial (LA) diameter was measured at end-diastole in the parasternal long-axis view [22].

### 2.2. Measurement of Apelin Levels

In our study, blood samples for apelin measurement were obtained only on the day of ICD implantation, during a standardized period when all patients were not volume overloaded, were not decompensated, and had remained in the supine position for at least one hour. Serum apelin levels were measured between 08:00 and 10:00 a.m. following an overnight fasting period of at least 12 h. Venous blood samples were preferably drawn from the left median cubital vein. The collected samples were centrifuged at 4000 rpm for 10 min, and the resulting serum was stored at −80 °C until analysis. After the completion of patient enrollment, all serum samples were analyzed simultaneously to ensure consistency.

Apelin measurements were successfully performed on all HFrEF and control patients included in the study. The laboratory performing the apelin measurements was blinded to the patients’ clinical information. Apelin levels were measured using a commercial enzyme-linked immunosorbent assay (ELISA) kit (E2014Hu; Bioassay Technology Laboratory, Shanghai Korain Biotech Co., Shanghai, China). This ELISA kit exhibits 100% cross-reactivity with human Apelin-12, Apelin-13, and Apelin-36 isoforms. The measurement range for apelin was 7–1500 ng/mL. The sensitivity of the assay was 0.00347 ng/mL (3.47 pg/mL). The intra-assay and inter-assay coefficients of variation (CV%) reported by the manufacturer were <8% and <10%, respectively.

### 2.3. Statistical Analysis

Variables were categorized as either categorical or continuous. Categorical variables were presented as frequencies and percentages, while continuous variables were expressed as mean ± standard deviation (SD). The kappa coefficient was used to assess inter-intraobserver variability for VT diagnosis and echocardiographic parameters. The normality of distribution for continuous variables was assessed using the Shapiro–Wilk test. Comparisons of continuous variables across three groups were performed using one-way ANOVA for normally distributed data or the Kruskal–Wallis one-way ANOVA for non-normally distributed data. For multiple comparisons of normally distributed variables, Scheffé or Games–Howell post hoc tests were applied, depending on the homogeneity of variances. For non-normally distributed variables, Bonferroni-adjusted Mann–Whitney U tests were used for pairwise comparisons. Fisher’s exact test was applied to evaluate differences in non-numeric categorical parameters. For comparisons between two groups, either Student’s *t*-test (for normally distributed data) or the Mann–Whitney U test (for non-normally distributed data) was used. Chi-square test was used to compare categorical variables. To identify independent predictors of ventricular tachycardia (VT), all variables found to be statistically significant (*p* < 0.05) in the univariate analysis were included in a multivariate logistic regression model. Receiver operating characteristic (ROC) curve analysis was performed for the parameters identified as being associated with VT in the multivariate logistic regression analysis. The area under the curve (AUC) with 95% confidence intervals and the sensitivity and specificity of selected cut-off values for discriminating VT were determined. All statistical analyses were performed using IBM SPSS Statistics version 25.0 (SPSS Inc., Chicago, IL, USA). A *p*-value of < 0.05 was considered statistically significant.

## 3. Result

All parameters of the patients included in the study were compared between Group I, Group II, and Group III. Parameters that independently identified patients with VT were identified. In our study, the numbers of patients with single-chamber ICD, dual-chamber ICD, and CRT-D devices were 6, 10, and 12, respectively. Cohen’s kappa values evaluating interobserver agreement were greater than 0.90 for VT diagnosis and for all echocardiographic parameters (*p* < 0.001).

### 3.1. Demographic, Clinical, Medical Treatment, and Laboratory Data of Patient Groups

The demographic, clinical, medical treatment, and laboratory characteristics of the patient groups are presented in Table 1. Upon evaluation of the demographic and clinical data, no statistically significant differences were observed among the three groups, indicating baseline comparability across the study population.

However, serum apelin levels were found to be significantly lower in both Group I and Group II compared with Group III (*p* < 0.05). In addition, apelin levels were found to be significantly lower in Group I compared with Group II (*p* < 0.05). Similarly, serum glucose and creatinine levels were also significantly elevated in Groups I and II compared with Group III, with no significant difference between Group I and Group II. Conversely, HDL cholesterol levels were significantly lower in Groups I and II compared with Group III, again with no significant difference between the HFrEF subgroups.

Regarding echocardiographic parameters, the LA diameter was significantly greater in Groups I and II compared with Group III (*p* < 0.05), while LVEF was significantly lower in Groups I and II compared with Group III. No significant differences in LA diameter or LVEF were observed between Group I and Group II.

In summary, serum apelin, glucose, creatinine, HDL cholesterol levels, as well as LA diameter and LVEF, differed significantly between the HFrEF patients (Groups I and II) and controls (Group III) but did not significantly differ between HFrEF patients with and without VT.

### 3.2. Parameters Associated with Serum Apelin Levels

When HFrEF patients were stratified according to NYHA class, no significant difference was found in plasma apelin levels across the NYHA stages (*p* = 0.306). The plasma apelin levels for NYHA I, II, III, and IV were 0.68 ± 0.37, 0.93 ± 0.46, 0.97 ± 0.50, and 0.93 ± 0.48, respectively.

Demographic, clinical, laboratory, and echocardiographic parameters associated with plasma apelin levels in univariate analysis are summarized in Table 2. Parameters showing a significant association with plasma apelin levels were included in a linear regression analysis (Table 2). Plasma apelin levels were found to be closely related to age and LVEF (Table 2).

### 3.3. Independent Parameters of Ventricular Tachycardia

**Figure 2:**
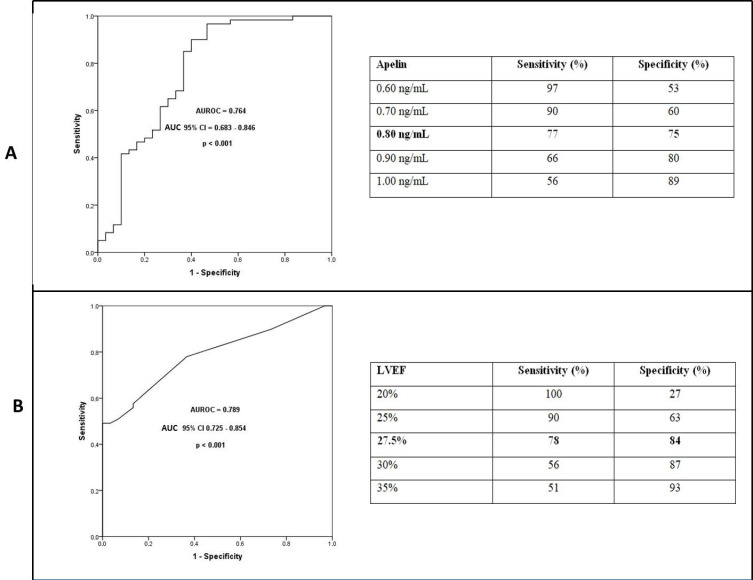
Receiver operating characteristic curves and cut-off levels for plasma apelin (**A**) and LVEF (**B**) in the prediction of ventricular tachycardia.

A multivariate logistic regression analysis was performed to identify variables closely and independently associated with VT. In the multivariate analysis, variables that differed between the groups in the univariate analysis—glucose, creatinine, high-density lipoprotein cholesterol, apelin, left ventricular ejection fraction, and left atrial dimension—were included. The results demonstrated that both LVEF and apelin levels were independently associated with the presence of VT (Table 3). In this model, each 1 ng/mL decrease in apelin level was associated with a 69% higher odds ratio of VT. Additionally, each 1% decrease in LVEF was associated with a 9% higher odds ratio of VT. In the ROC analysis conducted to evaluate the discriminative value of apelin and LVEF for identifying patients with VT, the area under the curve was found to be 0.764 for apelin and 0.789 for LVEF (Figure 2). Using a cut-off value of 0.80 ng/mL for apelin, the presence of VT could be discriminated with 77% sensitivity and 75% specificity. For LVEF, a cut-off value of 27.5% yielded 78% sensitivity and 84% specificity in discriminating VT.

## 4. Discussion

This study presents several important findings: (i) plasma apelin levels were found to be reduced in patients with HFrEF, which is consistent with the majority of previously published studies; (ii) decreased apelin levels were found to be independently associated with the presence of VT in HFrEF patients, a novel finding not previously demonstrated in the literature; and (iii) plasma apelin levels were positively associated with LVEF and negatively associated with age, also in agreement with prior studies.

One possible reason for the variability in apelin levels across different studies may be the heterogeneity of the patient populations. The largest study evaluating plasma apelin levels in HFrEF patients to date was conducted by Chong et al. [6], which included 202 HFrEF patients and showed that plasma apelin levels were significantly lower than those in the control group. In the same study, apelin levels were reported to be consistently reduced across all NYHA classes, with no significant variation by class [6]. In contrast, another study reported increased apelin levels in NYHA classes II and III, and a decrease in class IV [12]. Our findings support those of Chong et al., indicating no significant variation in apelin levels across NYHA classes. In the study by Foldes et al. [7], which included only HFrEF patients with ischemic etiology, plasma apelin levels were significantly reduced.

Previous studies have also reported associations between plasma apelin levels and several clinical and biochemical markers, including age, LVEF, hs-CRP, and LDL cholesterol [6,19]. In our study, we also observed a positive correlation with LVEF and a negative correlation with age, consistent with prior data.

Apelin exerts strong inotropic effects, promotes nitric-oxide-mediated vasodilation, reduces ventricular preload and afterload, and enhances coronary blood flow [23]. Furthermore, it plays a protective role in the development and progression of heart failure by antagonizing the renin–angiotensin–aldosterone system (RAAS) [24]. However, the activation of RAAS leads to downregulation of the apelinergic system, thereby diminishing the cardioprotective effects of apelin [25]. Thus, it is plausible that reduced apelin levels in HFrEF patients are linked to worse prognosis. Indeed, several studies have demonstrated the prognostic importance of decreased apelin levels in HFrEF [3,19].

In addition to HFrEF, reduced plasma apelin levels have also been reported in other cardiovascular diseases with poor prognoses, such as atrial fibrillation and aortic stenosis [26,27]. In a study by Liu HT et al. [19], lower apelin levels were found to be associated with adverse cardiovascular events in patients with ST-segment elevation myocardial infarction. That study proposed a threshold value of 0.54 ng/mL for plasma apelin as a prognostic marker. Although our study did not directly assess mortality or morbidity outcomes in HFrEF patients, it evaluated the presence of VT. We found that decreased apelin levels were independently associated with VT, and that each 1 ng/mL decrease in apelin levels increased the odds ratio of VT by 69%. Furthermore, a cut-off apelin level of 0.80 ng/mL discriminated VT with 77% sensitivity and 75% specificity. In line with the literature, we also observed a strong association between reduced LVEF and the presence of VT in HFrEF patients [22].

### 4.1. Clinical Implications

Apelin is a mechanistically interesting biomarker but requires validation before clinical use, particularly for arrhythmia risk stratification in HFrEF.

### 4.2. Limitation

This study has several limitations that should be acknowledged. First, it was a retrospective, single-center study conducted in a relatively small sample size. A prospective, multicenter study involving a larger population would provide more robust and generalizable findings. Second, the study included only patients with HFrEF. Including patients with HFpEF may have provided a more comprehensive understanding of apelin dynamics across different heart failure phenotypes. Another potential source of variability in apelin levels reported across heart failure studies is the type of medical therapy administered to HFrEF patients. Previous studies have shown that medications such as spironolactone, SGLT-2 inhibitors, and cardiac resynchronization therapy may increase plasma apelin levels [8,28,29]. In our study, all patients were treated according to the current guideline-directed medical therapy for HFrEF. However, the effects of specific medications on apelin levels were not analyzed, and this remains a potential confounding factor. The control group is described as “age- and sex-matched,” but controls appear to have significantly different creatinine, glucose, HDL, and LA size, suggesting imperfect matching. All of the above-mentioned variables may significantly influence not only the risk of VT but also the apelin levels. The patients included in our study were not selected to ensure equal distribution across NYHA classes; therefore, apelin levels may not have differed significantly according to NYHA class. Serum apelin levels have been reported to be an important prognostic parameter in cardiovascular diseases [19,26,27]. However, prognostic assessment was not performed in our study. Other limitations of our study include possible selection bias in an arrhythmia/ICD clinic population, residual confounding (such as GDMT intensity and device programming), and the absence of external validation for the ROC cut-off values.

## 5. Conclusions

First, our study is a pilot study designed to evaluate the hypothesis that apelin is associated with VT in patients with HFrEF. Based on the findings of our study, serum apelin levels were significantly reduced in patients with HFrEF, and lower apelin levels were associated with the presence of VT in this population. In light of the data obtained in our study, we believe that prospective, multicenter studies with larger patient populations are warranted to determine whether serum apelin levels can discriminate the presence of VT in patients with HFrEF.

## Figures and Tables

**Table 1:** Demographic, clinical, medical treatment, and laboratory findings of the study groups.

Variable	Group I *n* = 30	Group II *n* = 30	Group III *n* = 30	*p*
Age (year)	65.8 ± 10.6	64.3 ± 9.1	63.4 ± 10.9	0.278
Gender (female), *n*	4 (13)	6 (20)	5 (17)	0.567
Hypertension, *n* (%)	24 (80)	27 (90)	19 (63)	0.123
Diabetes mellitus, *n* (%)	13 (43)	16 (53)	7 (23)	0.116
Smoking, *n* (%)	21 (70)	20 (67)	19 (63)	0.435
Coronary artery disease history, *n* (%)	6 (20)	6 (20)	10 (33)	0.256
Previous cerebrovascular accident, *n* (%)	5 (17)	4 (13)	4 (13)	0.545
NYHA class I-II-III-IV, *n* (%)	4(13)-16(53)-9(30)-1(4)	5(17)-17(57)-7 (23)-1(4)	–	0.799
Heart rate (beat/min)	76 ± 7.8	77 ± 11	76 ± 11	0.167
Systolic blood pressure (mmHg)	122 ± 13	123 ± 11	121 ± 12	0.345
Diastolic blood pressure (mmHg)	74 ± 7.7	76 ± 6.8	76 ± 8.8	0.485
Body mass index (kg/m^2^)	27.8 ± 4.6	27.7 ± 3.8	28.1 ± 3.4	0.762
Beta blocker use, *n* (%)	30 (100) ^a^	30 (100) ^a^	18 (60) ^b^	<0.001
ARNI, ACEI or ARB use, *n* (%)	28 (93) ^a^	30 (100) ^a^	18 (60) ^b^	<0.001
MRA use, *n* (%)	12 (40) ^a^	9 (30) ^a^	0 (0) ^b^	0.002
SGLT2i use, *n* (%)	12 (40) ^a^	15 (50) ^a^	2 (7) ^b^	0.009
Amiodarone use, *n* (%)	8 (27) ^a^	5 (17) ^a^	0 (0) ^b^	0.003
Furosemide, *n* (%)	27 (90) ^a^	30 (100) ^a^	5 (17) ^b^	<0.001
ICD therapies, *n* (%)	30 (100) ^a^	0 (0)	–	<0.001
White blood cell (µL)	9.1 ± 2.7	8.6 ± 2.9	7.9 ± 2.5	0.256
Hemoglobin (g/dL)	13.2 ± 2.2	13.7 ± 1.7	13.1 ± 2.3	0.474
Glucose (mg/dL)	130 ± 42 ^α^	132 ± 37 ^β^	103 ± 21	**0.006**
Blood urea nitrogen (mg/dL)	48.1 ± 30.1	42.7 ± 29.8	31.9 ± 17.5	0.068
Creatinine (mg/dL)	1.04 ± 0.41 ^α^	1.03 ± 0.46 ^β^	0.76 ± 0.26	**0.009**
Total cholesterol (mg/dL)	186 ± 55	196 ± 58	202 ± 27	0.490
Low-density lipoprotein cholesterol (mg/dL)	120 ± 44	132 ± 41	132 ± 19	0.427
High-density lipoprotein cholesterol (mg/dL)	42.6 ± 8.6 ^α^	40.8 ± 9.4 ^β^	49.4 ± 9.9	**0.015**
Triglyceride (mg/dL)	176 ± 592	182 ± 102	173 ± 104	0.945
High-sensitivity C-reactive protein (mg/L)	20.4 ± 24.8	17.2 ± 28.5	5.9 ± 6.9	0.171
Apelin (ng/mL)	0.83 ± 0.55 ^¥,α^	1.02 ± 0.46 ^β^	1.77 ± 0.95	**0.001**
Left ventricular ejection fraction (%)	26.7 ± 6.8 ^α^	27.8 ± 5.3 ^β^	59.2 ± 3.3	**<0.001**
Left atrial dimension (mm)	42.6 ± 3.2 ^α^	41.1± 3.6 ^β^	34.3 ± 3.1	**<0.001**

The values were shown as mean ± standard deviation or n (%); Group I = heart failure with ventricular tachycardia, Group II = heart failure without ventricular tachycardia, and Group III = control group. ¥ = the significant association between the Group I and Group II (*p* < 0.05). α = the significant association between the Group I and Group III (*p* < 0.05). β = the significant association between the Group II and Group III *p* < 0.05). a = *p* > 0.05 between two groups, b = *p* < 0.05 between the other two groups. Statistically significant *p* values are shown in bold.

**Table 2:** The parameters associated with plasma apelin level and linear regression analysis for parameters significantly correlated with plasma apelin level.

Variable	Univariate Analyze		Multivariate Analyze		
	*p*	r	*p*	β	95% CI
Age (year)	**<0.001**	−0.289	**0.009**	−0.190	−5.140–−0.898
Heart rate (beat/min)	0.964	−0.005	0.493	−0.092	−2.457–2.347
Systolic blood pressure (mmHg)	0.701	0.041	0.634	0.086	−1.672–2.475
Diastolic blood pressure (mmHg)	0.905	0.013	0.663	−0.080	−3.045–3.435
Body mass index (kg/m^2^)	0.602	−0.057	0.533	−0.086	−8.455–4.931
Hemoglobin (g/dL)	0.596	−0.057	0.634	−0.072	−14.855–8.582
Glucose (mg/dL)	**0.009**	−0.195	0.125	−0.118	−0.720–0.025
Blood urea nitrogen (mg/dL)	0.409	−0.089	0.380	−0.261	−1.279–0.526
Creatinine (mg/dL)	0.525	−0.068	0.221	0.346	−79.348–40.795
Total cholesterol (mg/dL)	0.787	0.031	0.898	0.038	−0.464–0.611
Low-density lipoprotein cholesterol (mg/dL)	0.765	0.034	0.840	0.056	−0.618–0.838
High-density lipoprotein cholesterol (mg/dL)	0.277	0.124	0.783	−0.047	−1.229–4.229
Triglyceride (mg/dL)	0.862	0.020	0.939	−0.013	−0.249–0.296
High-sensitivity C-reactive protein (mg/L)	0.420	−0.099	0.750	−0.047	−1.817–0.766
Left atrial dimension (mm)	**<0.001**	−0.328	0.207	− 0.097	−13.547–−3.211
Left ventricular ejection fraction (%)	**<0.001**	0.348	**0.006**	0.271	1.040–3.891

$R_{Adjusted}^{2}=0.394$ and control group was excluded in correlation and linear regression analyses. Statistically significant *p* values are shown in bold.

**Table 3:** Multivariate logistic regression analysis for identifying patients with ventricular tachycardia.

Variable	Odds Ratio	95% CI	*p*
Apelin (for each 1 ng/mL increase)	0.313	0.124–0.788	**0.014**
Left ventricular ejection fraction (for each 1% increase)	0.912	0.877–0.968	**<0.001**

## Data Availability

The original contributions presented in this study are included in the article. Further inquiries can be directed at the corresponding author.
